# Cranioplasty after Decompressive Craniectomy (DC) in a Patient with Intracerebral Hemorrhage after SARS-CoV-2 Vaccination-Related Vaccine-Induced Thrombotic Thrombocytopenia (VITT)—Proposal of a Management Protocol for This Rare Pathological Condition

**DOI:** 10.3390/jcm13164778

**Published:** 2024-08-14

**Authors:** Lennard Spanehl, Uwe Walter, Thomas Thiele, Daniel Dubinski, Bedjan Behmanesh, Thomas M. Freiman, Matthias Wittstock, Patrick Schuss, Hartmut Vatter, Matthias Schneider, Florian Gessler, Sae-Yeon Won

**Affiliations:** 1 Department of Neurosurgery, Rostock University Medical Center, 18057 Rostock, Germany; daniel.dubinski@med.uni-rostock.de (D.D.); bedjan.behmanesh@med.uni-rostock.de (B.B.); neurochirurgie@med.uni-rostock.de (T.M.F.); florian.gessler@med.uni-rostock.de (F.G.); sae-yeon.won@med.uni-rostock.de (S.-Y.W.); 2 Department of Neurosurgery, Brigham and Women’s Hospital, Harvard Medical School, Boston, MA 02115, USA; 3 Department of Neurology, Rostock University Medical Center, 18057 Rostock, Germany; uwe.walter@med.uni-rostock.de (U.W.); matthias.wittstock@med.uni-rostock.de (M.W.); 4 Department of Transfusion Medicine, University Medicine Rostock, 18057 Rostock, Germany; thomas.thiele@med.uni-rostock.de; 5 Department of Neurosurgery, Unfallkrankenhaus Berlin, 12683 Berlin, Germany; 6 Department of Neurosurgery, University Hospital Bonn, 53127 Bonn, Germany; neurochirurgie@ukbonn.de (H.V.); matthias.schneider@ukbonn.de (M.S.)

**Keywords:** cerebral sinus and vein thrombosis (CVT), decompressive craniectomy (DC), intracerebral hemorrhage (ICH), COVID-19, vaccination, SARS-CoV-2, vaccine-induced immune thrombotic thrombocytopenia (VITT)

## Abstract

The COVID-19 (coronavirus disease) pandemic had a severe impact on public health worldwide. A rare but serious complication after administration of adenoviral vaccines against SARS-CoV-2 (AstraZeneca–Oxford and Johnson & Johnson) is vaccine-induced immune thrombotic thrombocytopenia and thrombosis (VITT), which can lead to serious complications such as cerebral venous sinus thrombosis (CVST). CVST itself can cause subarachnoid hemorrhage (SAH) and/or intracerebral hemorrhage (ICH), leading to high mortality due to herniation of brain parenchyma. In those patients, an emergent decompressive hemicraniectomy (DC) is regularly performed. Herein, the authors want to focus on the patients who survive DC following VITT-associated CVST and shed light on the neurosurgical considerations in those patients. We herein propose a treatment algorithm regarding the timing and the perioperative management of cranioplasty. We describe an exemplary case highlighting that special circumstances may result in a more urgent need for autologous cranioplasty than usual, based on individual risk assessment.

## 1. Introduction

The COVID-19 (coronavirus disease) pandemic had a severe impact on public health worldwide. Nearly 70% of the world population has received at least one dose of a COVID-19 vaccination [1]. While overall, these vaccinations saved millions of lives [2], a rare but serious complication that has been reported almost exclusively after administration of the adenoviral vaccines from AstraZeneca-Oxford and Johnson & Johnson is vaccine-induced immune thrombotic thrombocytopenia and thrombosis (VITT) [3]. This is a condition characterized by thrombosis, thrombocytopenia, hypofibrinogenemia, and elevated D-dimer concentration. The pathophysiology is still not fully understood but seems to have similarities with that of heparin-induced thrombocytopenia (HIT). One theory proposes a two-step model in which adenoviral vector vaccine components bind with platelet factor 4 (PF4), leading to neoantigen formation. In the second step, vaccine components trigger a proinflammatory response, which in turn causes a pronounced B-cell response with antibodies against PF4–polyanion complexes that cause platelet and neutrophil activation, causing thrombosis [4,5,6].

There are several different treatment options for patients presenting with CVST, such as direct endovascular thrombolysis [7], intravenous anticoagulation with heparin [8], and oral anticoagulation [9]. Patients with suspected VITT should be treated with non-heparin anticoagulants and intravenous immunoglobulin [10]. Herniation of brain parenchyma triggered by an ischemic or hemorrhagic stroke is the most common cause of death in patients with CVST [11]. A decompressive hemicraniectomy (DC) seems to be beneficial in terms of mortality and functional outcome in patients with severe CVST and large parenchymal lesions causing herniation [11,12]. Nevertheless, the prognosis is poor. In a large multicenter cohort study, the mortality rate for patients with VITT-associated CVST receiving decompressive hemicraniectomy was 54%. This is more than three times higher than the mortality for patients receiving DC because of non-VITT-associated CVST [10]. While extensive data exist on the medicinal treatment of VITT-associated CVST, data regarding DC in those patients are still limited, with no valid data on the optimal timing of cranioplasty in those patients.

Herein, the authors want to focus on the patients who survive DC following VITT-associated CVST and shed light on the neurosurgical considerations regarding cranioplasty in those patients. Based on a brief literature review, we will propose an extension of a previously published treatment algorithm and review an exemplary case in which we performed a cranioplasty after VITT-associated CVST and DC, also highlighting special considerations for patients with frequent seizures/falls. Since limited data exist on performing DC on VITT patients, to the best of our knowledge, our work is the first that focuses on cranioplasty in this patient population. Thus, we hope that our manuscript provides a practical guideline for clinicians to follow when treating patients with cranioplasty after VITT-associated CVST.

## 2. Literature Review

### 2.1. Epidemiology and Diagnosis 

Although VITT is relatively rare, with an incidence of approximately 1 per 100,000 vaccinations (ranging between 1 in 26,000 and 1 in 263,000 individuals) [13,14], the facts that adenoviral vector-based vaccines have also been developed for other diseases than COVID-19 and some evidence suggests that VITT might not be limited to anti-SARS-CoV-2 vaccines highlight the ongoing relevance of this topic [15]. VITT itself can lead to serious complications such as cerebral venous sinus thrombosis (CVST), which has been described as the most common site of thrombosis in previous studies [6,16,17]. The incidence of CVST after administration of the AstraZeneca–Oxford vaccine is about 10-fold higher than in the general population, and the patients affected are younger than those with non-VITT-associated CVST. In addition, a large multicenter study from the UK observed that CVST is more extensive in VITT patients than in patients without VITT, with increased mortality in those patients [10]. In a systematic review, 49% of patients with VITT-associated CVST developed subarachnoid hemorrhage (SAH) and/or intracerebral hemorrhage (ICH), leading to a mortality rate of 39% [17]. The diagnosis of a CVST is typically made by CT or MR venography of the brain [3]. The diagnosis of VITT is multimodal and typically confirmed if the following criteria are met: onset of symptoms 5–30 days after vaccination against SARS-CoV-2; presence of thrombosis; thrombocytopenia (platelet count < 150,000 per cubic millimeter); d-dimer level > 4000 FEU; positive anti-PF4 antibodies on ELISA [18]. It should be noted that VITT may rarely occur without evident thrombocytopenia [19].

Initial outcomes of patients with VITT-associated CVST were poor [10]. The reported mortality ranges between 47% and 22% [20]. Previous studies have demonstrated DC as a surgical method in the case of CVST to be a life-saving measure [11,12]. The following study is the first study concentrating on the optimal timing of cranioplasty in the specific cohort of patients with VITT-associated CVST who underwent DC. 

### 2.2. Initial Pharmacological Treatment of VITT-Associated CVST 

It is recommended to immediately start treatment with IVIg at 1 g/kg per day for two days, avoid platelet transfusions, and start therapeutic-dose non-heparin anticoagulation in VITT patients [3]. IVIg was associated with a significant reduction in mortality in VITT patients with CVST [21]. In patients with severe thrombocytopenia and CVST, the use of therapeutic plasma exchange can be considered [18]. The recommendation of using non-heparin anticoagulation stemmed from similarities between VITT and HIT. However, meanwhile, meta-analyses show no increased mortality in VITT patients treated with heparin anticoagulants compared to non-heparin-based anticoagulants, and it is known that the pathophysiology of HIT and VITT differs [22]. In patients with CVST, the anticoagulation should initially be administered parenterally with a switch to direct oral anticoagulants in the subacute/chronic phase [3]. The reason why anticoagulation should even be used in patients with ICH secondary to CVST is [18] that the ICH is caused by venous stasis in those patients and often resolves with anticoagulation. That is why progressive thrombosis must be prevented [23,24]. However, an expanding ICH is a contraindication for therapeutic anticoagulation. Ultimately, anticoagulation in patients with ICH remains a double-edged sword, as the risk of progressive bleeding must be compared with the risks of progressive thrombosis. 

### 2.3. Duration of Anticoagulation

While extensive data exist on the initial management of VITT, to date, the optimal follow-up of patients with VITT is still under debate. Particularly, determining the optimal duration of anticoagulation remains challenging [23,25,26]. Even though anti-PF4 IgG antibodies seem to persist for a long time (as indicated by positive anti-PF4-heparin IgG ELISA), the majority of platelet-activating antibodies seem to disappear within 12 weeks (as indicated by negative platelet activation assays) [27]. According to several guidelines, anticoagulation in patients with VITT should be continued for at least 3 months and may be needed even longer for patients with CVST [28]. While some authors propose the simple approach, that anticoagulation should be continued for 3 months after normalization of the platelet count in VITT patients with thrombosis [29], others state that PF4 antibodies should disappear prior to discontinuation of anticoagulation [30]. In this context, platelet functional testing using a PF4-induced platelet activation assay (PIPA) might help determine the duration of anticoagulation [23]. We prefer discontinuation of anticoagulation after the proven disappearance of serum anti-PF4 antibodies. After stopping the anticoagulation, platelet count and serum D-dimer levels should be controlled weekly for another 4 weeks to ensure the complete remission of VITT.

### 2.4. Reasons and Timing of Cranioplasty

The reasons for performing a cranioplasty are not only a cosmetic restoration or improvement of cerebral protection but also positive effects on cerebral blood flow and glucose metabolism (and thereby an improvement of the neurologic outcome) [31]. In general, cranioplasty should be performed in patients who are in a stable condition after brain swelling has resolved [32]. While it was proposed that early cranioplasty seems to improve patients’ neurological functions [33], according to a retrospective study on patients who received cranioplasty after DC because of supratentorial cerebral infarction, a late cranioplasty (>3 months) was associated with significantly lower complication rates (42% complication rate in patients receiving cranioplasty within 3 months after DC vs. 13% in patients treated after 3 months). In particular, wound healing appears to be improved when delaying cranioplasty [32]. The complication rate was at the lowest level for patients receiving cranioplasty within 3–6 months after DC [32]. Additionally, the aforementioned study found no significant differences in neurologic functional outcomes between the early and late cranioplasty groups. Weighing the possible improvements in the neurological outcomes of a heterogeneous patient cohort against the increased complication rate in early surgery, an adequate time frame for cranioplasty might be between 2 and 6 months after DC [34]. 

## 3. Proposal of a Treatment Algorithm for Cranioplasty in Patients with VITT-Associated CVST with Special Considerations, e.g., Frequent Seizures/Falls

**Scheme 1:**
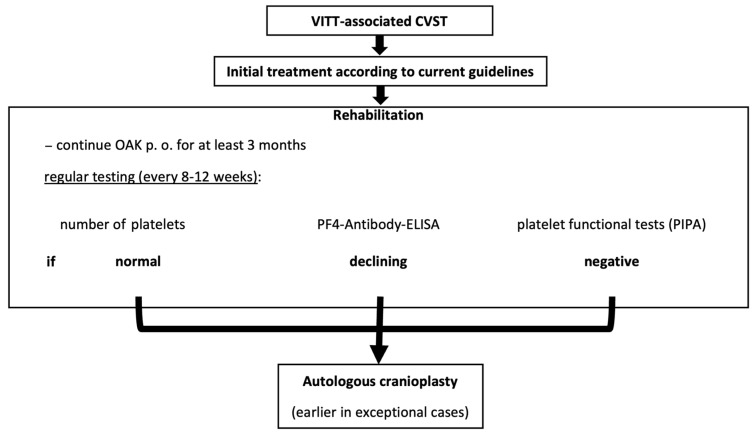
Treatment algorithm for cranioplasty in patients surviving DC after VITT-associated CVST.

In a previous study, we summarized a neurosurgical treatment algorithm for patients presenting with a VITT-associated CVST, stating that some patients need an emergent DC [35]. Even though the prognosis is poor in these patients, for those who survive, there is no data regarding the timing of cranioplasty. As mentioned above, an adequate time frame for cranioplasty might be between 2 and 6 months after DC [34]. To minimize the periprocedural bleeding risk, we propose that, in general, the cranioplasty should be performed after discontinuation of the anticoagulation. While the optimal duration of anticoagulation is still under debate, after 3 months of anticoagulation, we recommend regular testing (every 8–12 weeks) of the number of platelets as well as PF4 antibody ELISA and platelet functional tests (PIPA). A negative PIPA test accompanied by a decrease in PF4 antibodies should then lead to discontinuation of anticoagulation if there are no other indications for a continuation. Cranioplasty can be planned elective afterwards. However, in exceptional cases, such as patients who need cerebral protection due to frequent seizures/falls, earlier surgery may be necessary (see below). Scheme 1 depicts our proposed addition of cranioplasty for the previously published treatment algorithm for patients surviving DC after VITT-associated CVST [35].

## 4. Exemplary Case Presentation

**Figure 1:**
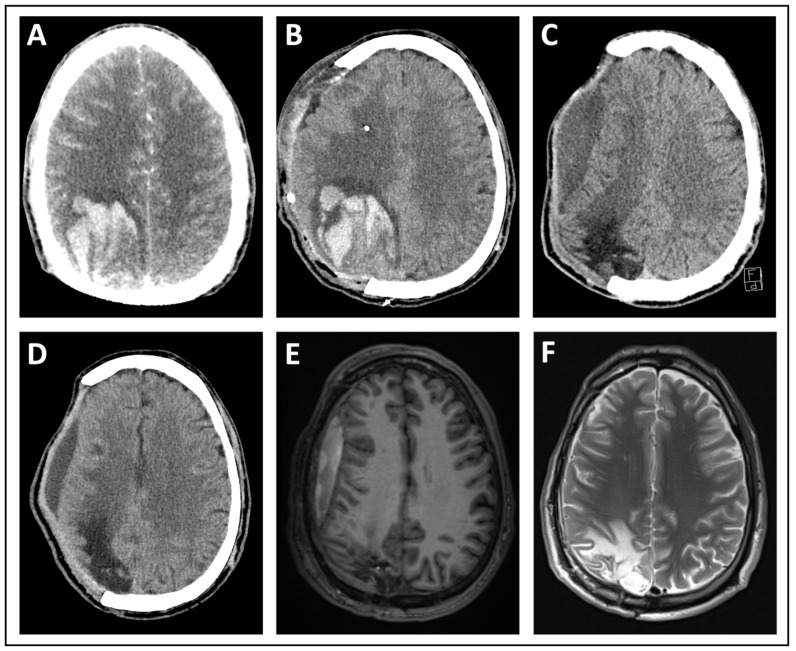
(**A**) Preoperative contrast-enhanced CT scan which demonstrates parenchymal stasis hemorrhage and thrombosis of the superior sagittal sinus. (**B**) CT scan immediately after decompressive hemicraniectomy (DC) and implantation of intracranial pressure sonde right frontal for ICP monitoring. (**C**) CT scan which shows chronic subdural hematoma (cSDH) 4.5 months after DC. (**D**) CT scan which shows cSDH before cranioplasty. (**E**) Postoperative T1-weighted MRI after cranioplasty. (**F**) T2-weighted MRI 8 months after cranioplasty.

Herein, the authors present a neurosurgical perspective on the management of a patient who received DC after VITT-associated CVST (see Table 1 for patient characteristics). The case of this patient was described in our previous paper regarding early neurosurgical treatment considerations in VITT patients with CVST [35]. The patient presented to the Emergency Department with nausea, emesis, headaches, and visual disturbances 12 days after the first vaccination with the Ad26.COV2.S vaccine (JNJ-78436735) for SARS-CoV-2. On examination, the patient presented with left-sided hemiparesis, homonymous hemianopsia to the left, and gait unsteadiness. Head computed tomography imaging, including a venogram, revealed thrombosis of the superior sagittal sinus and ICH (Figure 1A) with beginning hydrocephalus (not visible in these layers). Laboratory values on admission demonstrated a substantial thrombocytopenia (i.e., platelet count of 72 G/L).

Given that VITT was suspected, therapy with intravenous immunoglobulin (IVIg at 1 g/kg body weight/d) and argatroban i. v. (targeted-aPTT: 50–70 s) was immediately initiated after consultation with the hematology department [3]. 

Administration of heparin and platelet transfusions were avoided [36]. The patient suffered neurological deterioration within a few hours with a decrease in vigilance and dilated pupils on the right side, warranting emergent surgical intervention, i.e., DC (Figure 1B). The bone flap was frozen and stored under sterile conditions at −80 °C immediately after DC. After surgery, the pupils appeared equal and promptly responsive to light. The patient was transferred to the intensive care unit postoperatively. The patient was extubated on day 14 after surgery. On day 18 after surgery, the patient could be discharged to a neurological rehabilitation center in an awake, partially oriented state with residual hemiparesis and sensory aphasia. The anticoagulation was switched to dabigatran p. o. [3].

At our institution, cranioplasty is typically planned elective after 3 months [34]. However, at this time, the patient was still anticoagulated with dabigatran. Anticoagulation could not be paused because platelet-reactive autoantibodies were still detectable (strongly positive PIPA test). To minimize the risk of bleeding, cranioplasty was postponed, and the abovementioned laboratory tests were scheduled at 4-week intervals. However, in the meantime, the patient suffered from a seizure, leading to a fall at 4.5 months after surgery. This resulted in a chronic subdural hematoma, which was initially managed conservatively (Figure 1C,D). After weighing the risk of delayed cranioplasty against the increased bleeding risk of surgery under anticoagulation, we decided to perform the procedure despite the need for anticoagulation. The reason for this procedure was the risk of brain parenchyma damage because of the tendency to fall during frequent seizures. Autologous cranioplasty was performed 6 months after surgery, even though oral anticoagulation with dabigatran was still necessary (positive PIPA test) (Figure 1E). The anticoagulation was switched from dabigatran to danaparoid (2 × 750 IU) for the operation. The postoperative CT scan showed no signs of re-bleeding. The patient was discharged to further neurological rehabilitation in good clinical condition. Anticoagulation was switched to dabigatran after suture removal. The level of serum anti-PF4 antibodies was controlled thereafter every 3 months using the PIPA test [5], and a slow drop in antibody levels was found, with a still significant level detected 10 months after vaccination. Thirteen months after the DC, the patient was in a stable clinical state; the PIPA test was negative for the first time, and oral anticoagulation was discontinued.

## 5. Conclusions

Special circumstances such as threatening brain parenchyma damage due to frequent seizures/falls may result in a more urgent need for autologous cranioplasty than usual. In such cases, the increased bleeding risk of the procedure under anticoagulation must be weighed against the risks of a delayed procedure on a patient-specific basis. Even though our current evidence in this selected patient population is limited, with our article only reporting the outcome of one patient, we do believe that for most patients surviving DC after VITT-associated CVST, our proposed treatment algorithm is reasonable according to the current literature. Additionally, to the best of our knowledge, this is the first report of a cranioplasty performed in a patient who underwent DC after VITT-associated CVST.

## Figures and Tables

**Table 1:** Patient characteristics.

Characteristic	
SARS-CoV-2 vaccine	Ad26.COV2.S
age (years)	29
sex	male
medical history	-
medication prior to ictus	-
time from vaccination to admission (days)	12
time between DC and cranioplasty (months)	6

## Data Availability

Data may be available upon reasonable request.

## Abbreviations

aPTT	activated partial thromboplastin time
COVID-19	coronavirus disease
cSDH	chronic subdural hematoma
CT	computed tomography
CVST	cerebral venous sinus thrombosis
DC	decompressive hemicraniectomy
ELISA	enzyme-linked immunosorbent assay
HIT	heparin-induced thrombocytopenia
ICH	intracerebral hemorrhage
ICP	intracranial pressure
IVIg	intravenous immunoglobulin
MR	magnetic resonance
MRI	magnetic resonance imaging
PF4	platelet factor 4
PIPA	PF4-induced platelet activation assay
SAH	subarachnoid hemorrhage
SARS-CoV-2	severe acute respiratory syndrome coronavirus 2
VITT	vaccine-induced immune thrombotic thrombocytopenia and thrombosis
